# The ABC-Type Efflux Pump MacAB Is Involved in Protection of Serratia marcescens against Aminoglycoside Antibiotics, Polymyxins, and Oxidative Stress

**DOI:** 10.1128/mSphere.00033-21

**Published:** 2021-03-10

**Authors:** Tatiana V. Shirshikova, Cecilia G. Sierra-Bakhshi, Leisan K. Kamaletdinova, Lilia E. Matrosova, Nailya N. Khabipova, Vladimir G. Evtugyn, Irina V. Khilyas, Iuliia V. Danilova, Ayslu M. Mardanova, Margarita R. Sharipova, Lydia M. Bogomolnaya

**Affiliations:** a Department of Microbiology, Institute of Fundamental Medicine and Biology, Kazan (Volga Region) Federal University, Kazan, Russia; b Interdisciplinary Center for Analytical Microscopy, Kazan (Volga Region) Federal University, Kazan, Russia; c Department of Biomedical Sciences, Joan C. Edwards School of Medicine, Marshall University, Huntington, West Virginia, USA; Escola Paulista de Medicina/Universidade Federal de São Paulo

**Keywords:** *Serratia marcescens*, MacAB, efflux pump, antibiotic resistance, physiological role, *Serratia*, multidrug efflux pumps, oxidative stress

## Abstract

Serratia marcescens is an emerging pathogen with increasing clinical importance due to its intrinsic resistance to several classes of antibiotics. The chromosomally encoded drug efflux pumps contribute to antibiotic resistance and represent a major challenge for the treatment of bacterial infections. The ABC-type efflux pump MacAB was previously linked to macrolide resistance in Escherichia coli and Salmonella enterica serovar Typhimurium. The role of the MacAB homolog in antibiotic resistance of S. marcescens is currently unknown. We found that an S. marcescens mutant lacking the MacAB pump did not show increased sensitivity to the macrolide antibiotic erythromycin but was significantly more sensitive to aminoglycoside antibiotics and polymyxins. We also showed that, in addition to its role in drug efflux, the MacAB efflux pump is required for swimming motility and biofilm formation. We propose that the motility defect of the *ΔmacAB* mutant is due, at least in part, to the loss of functional flagella on the bacterial surface. Furthermore, we found that the promoter of the MacAB efflux pump was active during the initial hours of growth in laboratory medium and that its activity was further elevated in the presence of hydrogen peroxide. Finally, we demonstrate a complete loss of *ΔmacAB* mutant viability in the presence of peroxide, which is fully restored by complementation. Thus, the S. marcescens MacAB efflux pump is essential for survival during oxidative stress and is involved in protection from polymyxins and aminoglycoside antibiotics.

**IMPORTANCE** The opportunistic pathogen Serratia marcescens can cause urinary tract infections, respiratory infections, meningitis, and sepsis in immunocompromised individuals. These infections are challenging to treat due to the intrinsic resistance of S. marcescens to an extensive array of antibiotics. Efflux pumps play a crucial role in protection of bacteria from antimicrobials. The MacAB efflux pump, previously linked to efflux of macrolides in Escherichia coli and protection from oxidative stress in Salmonella enterica serovar Typhimurium, is not characterized in S. marcescens. We show the role of the MacAB efflux pump in S. marcescens protection from aminoglycoside antibiotics and polymyxins, modulation of bacterial motility, and biofilm formation, and we illustrate the essential role for this pump in bacterial survival during oxidative stress. Our findings make the MacAB efflux pump an attractive target for inhibition to gain control over S. marcescens infections.

## INTRODUCTION

The emergence of bacterial multidrug resistance is a global public health concern worldwide. A number of Gram-negative enterobacteria, including Serratia marcescens, an opportunistic pathogen associated with endocarditis, osteomyelitis, septicemia, wound infections, and urinary and respiratory tract infections (1), were recognized as a serious threat by the World Health Organization (WHO) and were included in the list of priority pathogens with the urgent need for new antimicrobials (2). Bacteria utilize different mechanisms to protect themselves from antibiotics, including posttranslational or mutational changes in antibiotic targets, inactivation of antibiotics by hydrolysis or by transfer of a chemical group, reduced permeability to prevent the access of antibiotics to their target, and increased efflux through efflux pumps and porins (3). Bacterial efflux pumps are active transporters that mediate resistance to a broad range of structurally diverse antibiotics (4). In addition to drug export, efflux pumps are also involved in a number of physiological processes, including extrusion of toxic metabolites, siderophores, and quorum sensing molecules, modulation of motility and biofilm formation, and virulence (5–11).

Based on their structure and energy source, bacterial efflux pumps are classified into the following six major families: ABC (ATP-binding cassette) superfamily, RND (resistance-nodulation-division) family, SMR (small multidrug resistance) family, MFS (major facilitator superfamily), MATE (multidrug and toxic compound extrusion) family, and PACE (proteobacterial antimicrobial compound efflux) family (12). In S. marcescens, efflux pumps are currently not fully characterized. To date, six out of eight putative S. marcescens RND efflux pumps have been linked with multidrug resistance (13–17). In addition, the SMR-type pump SsmE and the MFS-type pump SmfY were shown to be involved in protection of S. marcescens from fluoroquinolones (18, 19). Finally, an ABC-type efflux pump, SmdAB, similar to VcaM from Vibrio cholerae, protects S. marcescens from fluoroquinolones and tetracycline (20). Analysis of the S. marcescens Db11 genome identified the presence of genes with high homology to *macAB* genes in E. coli K-12 (21). The MacAB efflux pump was first identified in E. coli, where it was linked to macrolide resistance (22), and later, was shown to have close homologs in other bacterial species (10, 23–26). In Salmonella enterica serovar Typhimurium, the MacAB efflux pump is required for full virulence in mice (10) and protection from oxidative stress (27, 28). The role of the MacAB efflux pump in antibiotic resistance and in the physiology of S. marcescens is currently unknown.

Here, we show that the MacAB efflux pump is not involved in the protection of S. marcescens from macrolide antibiotics but, instead, protects bacteria from clinically relevant aminoglycosides and contributes to the intrinsic resistance to polymyxins. We also show that in addition to its role in antibiotic resistance, this pump is involved in the modulation of motility and biofilm formation. We characterize conditions that lead to *macAB* promoter activation and demonstrate further activation of promoter by hydrogen peroxide. Finally, we show that the MacAB efflux pump is essential for the survival of S. marcescens during oxidative stress. We conclude that the MacAB efflux pump plays an important role in protection of S. marcescens from aminoglycoside antibiotics, from polymyxins, and from peroxide-mediated killing.

## RESULTS

### MacAB drug efflux pump in Serratia marcescens is not involved in the protection of bacteria against erythromycin.

**FIG 1:**
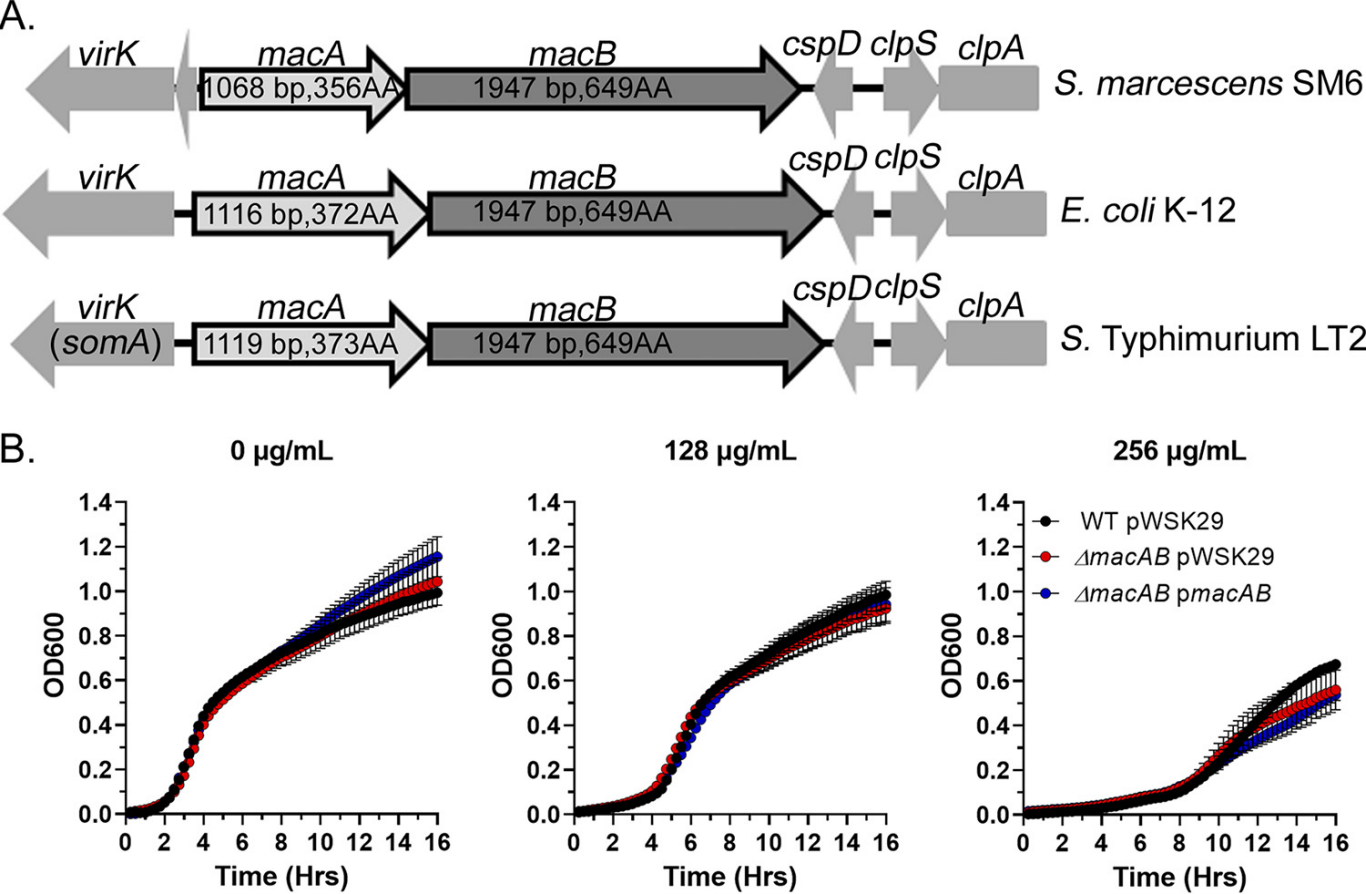
The MacAB drug efflux pump is not involved in protection of S. marcescens from macrolides. (A) Genomic context of the *macAB* locus in S. marcescens SM6 genome in comparison with E. coli K-12 and Salmonella enterica serovar Typhimurium LT2. (B to D). Growth curve of wild-type (black circles), Δ*macAB* mutant (red circles), and Δ*macAB* p*macAB* mutant (blue circles) strains of S. marcescens in the presence of 0 μg/ml (B), 128 μg/ml (C), or 256 μg/ml (D) of erythromycin. Data represent the means from at least three independent experiments and a standard error.

The genome of the opportunistic pathogen Serratia marcescens SM6 (29) contains a locus that is homologous to the *macAB* operon (EG355_04710-EG355_04715) present in E. coli and *Salmonella* Typhimurium (10, 22). S. marcescens periplasmic adaptor protein MacA is slightly smaller than the corresponding proteins in E. coli and *S*. Typhimurium (Fig. 1A) and shares 68 to 70% amino acid identity and 81 to 82% similarity to E. coli and *S.* Typhimurium MacA proteins. The MacB transporter protein in S. marcescens shares 72% identity and 84% similarity to the corresponding proteins in E. coli and *S.* Typhimurium and has the same overall polypeptide length as its homologs in both bacterial species.

The MacAB efflux system was previously linked to macrolide resistance in E. coli (22, 30). We hypothesized that the MacAB drug efflux pump in S. marcescens plays a similar role in protection against macrolide antibiotics. The growth of wild-type S. marcescens was significantly delayed by addition of erythromycin. Unexpectedly, deletion of the *macAB* locus in S. marcescens did not have any effect on bacterial growth in the presence of erythromycin (Fig. 1B to D). We conclude that the MacAB drug efflux pump is not involved in the protection of S. marcescens against macrolide antibiotics.

### MacAB is involved in protection of S. marcescens against aminoglycoside antibiotics.

To fully determine the impact of the MacAB efflux pump on S. marcescens antibiotic sensitivity, we used the commercially available Sensititre plates designed to determine MICs of different antibiotics (GN2F; Thermo Scientific). In agreement with previous reports (1), we found that the wild-type strain S. marcescens SM6 is resistant to several β-lactam and cephalosporin antibiotics based on the CLSI M100-Ed30 2020 performance standards for antimicrobial testing for *Enterobacterales* (31), such as ampicillin, ampicillin/sulbactam (2:1 ratio), cefazolin, cefoxitin, cefpodoxime, and cefuroxime (Table 1).

**TABLE 1:** MICs for Serratia marcescens SM6 wild type and *ΔmacAB* mutant

Antibiotic class	Antibiotic	Susceptibility criteria (MIC, mg/liter)^*a*^		MIC (mg/liter) for:^*b*^		
		S	R	Wild type	*ΔmacAB*	Complement
Aminoglycosides	Amikacin	≤16	≥64	**>64**	**8**	**≥32**
	Gentamicin	≤4	≥16	2	2	4
	Tobramycin	≤4	≥16	4	4	>8
Fluoroquinolones	Ciprofloxacin	≤0.25	≥1	0.5	1	0.5
	Gatifloxacin	≤2	≥8	1	2	1
β-Lactams	Ampicillin	≤8	≥32	>32	>32	>32
	Ampicillin/sulbactam (2:1 ratio)	≤8/4	≥32/16	>32/16	>32/16	>32/16
	Piperacillin	≤16	≥128	16	16	16
	Piperacillin/tazobactam constant 4	≤16/4	≥128/4	≥32/4	16/4	16/4
	Ticarcillin/clavulanic acid constant 2	≤16/2	≥128/2	16/2	≥32/2	≥32/2
Monobactams	Aztreonam	≤4	≥16	8	8	8
Carbapenems	Imipenem	≤1	≥4	2	2	2
	Meropenem	≤1	≥4	1	1	1
Nitrofuran derivatives	Nitrofurantoin	≤32	≥128	>128	>128	>128
Antifolates	Trimethoprim/sulfamethoxazole	≤2/38	≥4/76	0.5/9.5	1/19	1/19
Cephalosporins	Cefazolin	≤16	≥32	>32	>32	>32
	Cefepime	≤2	≥16	4	4	4
	Cefotetan Na	≤16	≥64	>16	16	8
	Ceftriaxone	≤1	≥4	1	1	1
	Ceftazidime	≤4	≥16	1	1	1
	Cefoxitin	≤8	≥32	≥32	>32	>32
	Cefpodoxime	≤2	≥8	8	8	8
	Cefuroxime	≤4	≥32	>32	>32	>32

a MIC susceptibility was determined based on the CLSI M100-Ed30:2020 performance standards for antimicrobial testing for *Enterobacterales*. S, susceptible; R, resistant.

b Bold text indicates a change in MICs between *S. marcescens* WT and the *macAB* mutant strains that is reverted by complementation.

**FIG 2:**
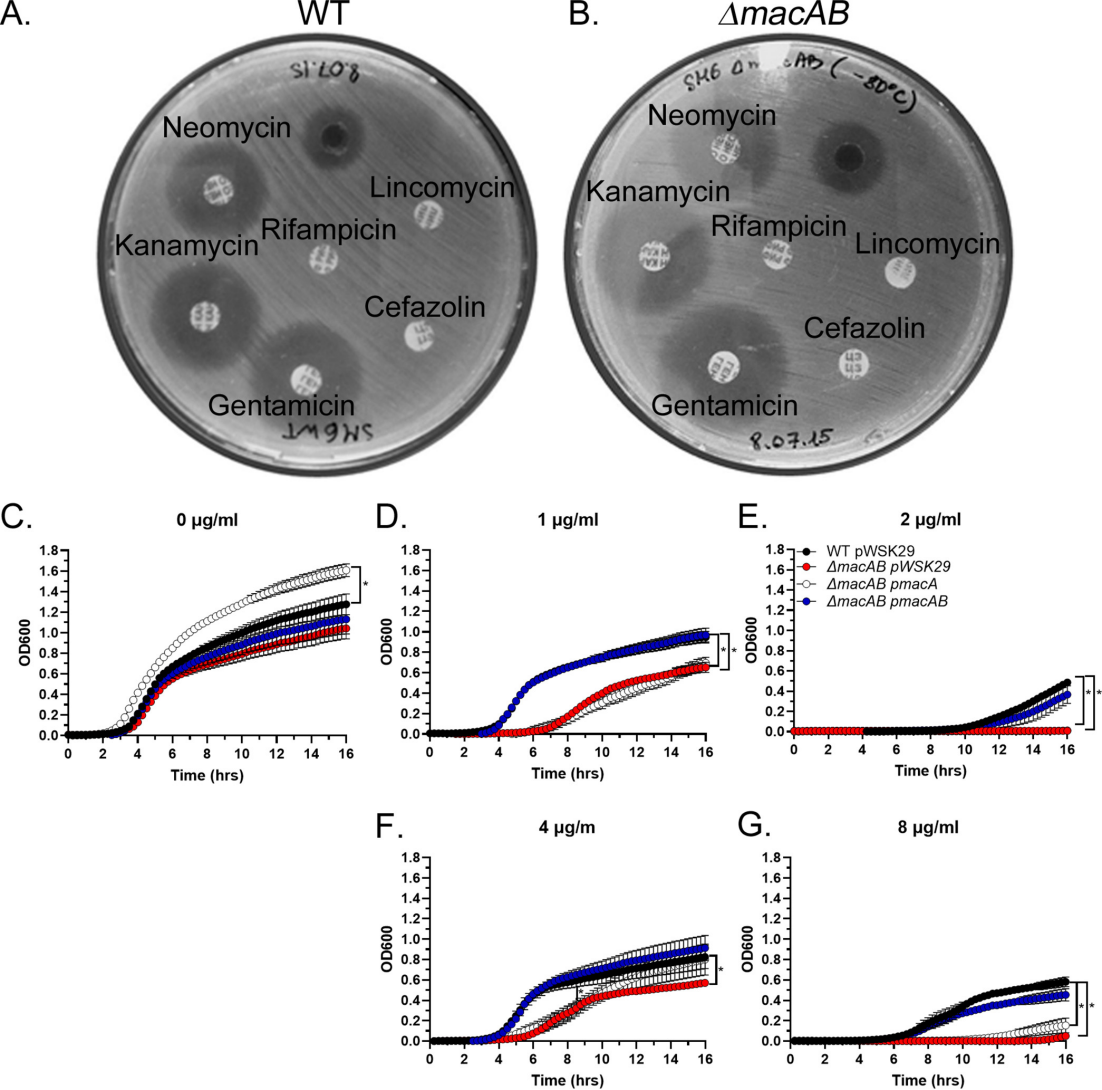
The Serratia marcescens MacAB efflux pump is required for protection from aminoglycosides. (A and G). Antibiotic susceptibility of wild-type (A) and *ΔmacAB* (B) strains was evaluated on Mueller-Hinton agar by disk diffusion assay. Disks contained antibiotics in the following concentrations: 30 μg neomycin, 30 μg kanamycin, 10 μg gentamicin, 5 μg rifampicin, 30 μg cefazolin, 15 μg lincomycin. (C to G). Growth of wild-type, Δ*macAB*, Δ*macAB* p*macA*, and Δ*macAB* p*macAB* strains in MH broth containing no antibiotic (C), 1 μg/ml (D) or 2 μg/ml gentamicin (E), and 4 μg/ml (F) or 8 μg/ml apramycin (G). Data represent the means from at least three independent experiments and a standard error. The asterisks indicate significance in an unpaired *t* test; *P < *0.05.

Until the mid-1970s, aminoglycoside antibiotics were the single drug of choice for the treatment of *Serratia* infections, and currently, they are used in combination with other antibiotics (32). We found that the S. marcescens SM6 wild-type strain is resistant to the aminoglycoside antibiotic amikacin but sensitive to gentamicin (Table 1). Furthermore, the loss of the MacAB drug efflux pump made S. marcescens sensitive to amikacin (Table 1). Because the Sensititre GN2F plates contain aminoglycoside antibiotics in a preset range of concentrations (8 to 64 μg/ml for amikacin and 2 to 16 μg/ml for gentamicin), we sought to confirm our results using the disk diffusion assay. In agreement with the Sensititre GN2F panel results (Table 1) and with previously published data (33), both wild-type and Δ*macAB* mutant strains were resistant to cefazolin, rifampicin, and lincomycin (Fig. 2A and B). However, the S. marcescens Δ*macAB* mutant strain was more sensitive to several aminoglycoside antibiotics, such as neomycin, kanamycin, and gentamicin, than to the isogenic wild-type strain (Fig. 2A and B and Table 2). Furthermore, addition of gentamicin to Mueller-Hinton (MH) broth in a range of concentrations from 0.5 to 2 μg/ml inhibited the growth of the Δ*macAB* mutant (red circles) compared to that of the wild-type strain (black circles) in a dose-dependent manner (Fig. 2C to E). Restoration of gentamicin resistance in the Δ*macAB* mutant strain required the presence of genes encoding both components of the efflux pump (Fig. 2C to E, blue circles), while introduction of the *macA* gene alone did not change drug sensitivity back to the wild-type level (Fig. 2C to E, white circles). To further confirm our findings, we tested the sensitivity of the Δ*macAB* mutant to another aminoglycoside antibiotic, apramycin. Similar to the situation with gentamicin, addition of apramycin to MH broth in the range of concentrations from 4 to 8 μg/ml resulted in statistically significant growth inhibition of the Δ*macAB* mutant in a dose-dependent manner compared to the wild-type strain. Growth was restored when plasmid-borne *macAB* genes, but not *macA* gene alone, were added to the Δ*macAB* mutant strain (Fig. 2F and G). Collectively, these data indicate that the MacAB efflux pump is involved in the protection of S. marcescens against aminoglycoside antibiotics.

**TABLE 2:** Serratia marcescens SM6 aminoglycoside susceptibility

Antibiotic	Disk content (μg)	Diam of inhibition zone (mm) for:	
		WT	*ΔmacAB*
Neomycin	30	18.0 ± 1.09	20.5 ± 1.52^*a*^
Kanamycin	30	20.0 ± 0.82	21.3 ± 0.50^*a*^
Gentamicin	10	20.5 ± 0.58	22.8 ± 1.26^*a*^

a Significance in two-tailed Student’s *t* test; *P *< 0.05.

### MacAB contributes to the intrinsic resistance to polymyxins in S. marcescens.

**FIG 3:**
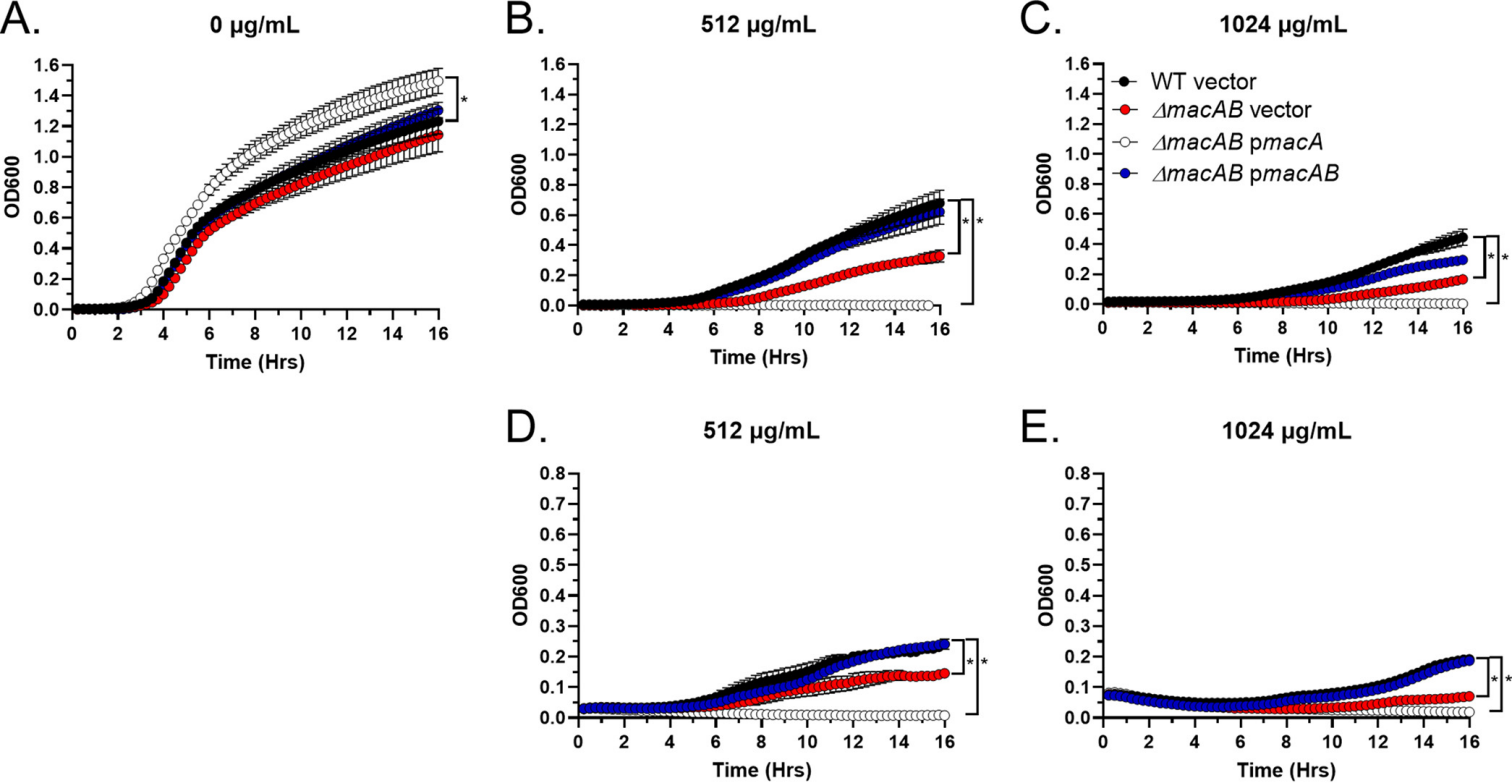
Serratia marcescens MacAB is involved in protection from polymyxins. (A to E) Growth of wild-type, Δ*macAB*, Δ*macAB* p*macA*, and Δ*macAB* p*macAB* strains in MH broth containing no antibiotic (A), 512 μg/ml (B) or 1,024 μg/ml (C) colistin, or 512 μg/ml (D) or 1,024 μg/ml (E) polymyxin B. Data represent the means from at least three independent experiments and a standard error. The asterisks indicate significance in unpaired *t* test, *P < *0.05.

Unlike many enterobacteria, S. marcescens is naturally resistant to polymyxins (34). This intrinsic resistance is based on PhoP-controlled expression of the *arnBCADTEF* operon, which results in modification of lipopolysaccharide (LPS) with aminoarabinose (35). Because the MacAB efflux pump is controlled by PhoP in *S.* Typhimurium (10, 30, 36), we sought to evaluate the impact of this pump on S. marcescens resistance to polymyxins. As expected, the wild-type strain was highly resistant to both colistin (polymyxin E) and polymyxin B (Fig. 3). Deletion of the *macAB* genes increased sensitivity of the mutant strain to both colistin and polymyxin B compared to the wild-type strain (Fig. 3). Resistance to polymyxins was fully restored by complementation of the Δ*macAB* mutant strain with plasmid-borne *macAB* genes (Fig. 3B to E, blue circles), but not with *macA* alone encoding the periplasmic adaptor protein (Fig. 3B to E, white circles).

These results indicate that the MacAB pump contributes to the intrinsic resistance of S. marcescens to polymyxins.

### MacAB plays a role in S. marcescens motility and biofilm formation.

**FIG 4:**
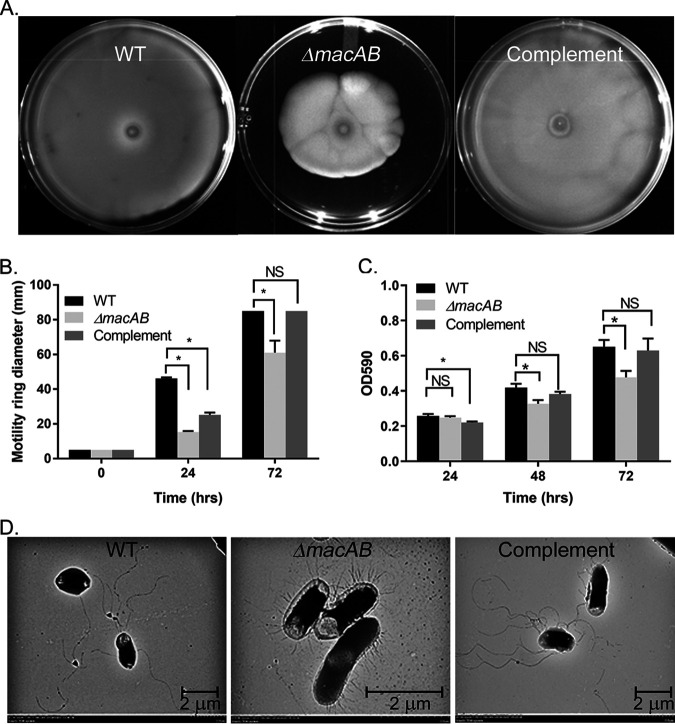
S. marcescens MacAB plays a role in bacterial motility and biofilm formation. Overnight cultures of the wild type, Δ*macAB* mutant strain, and Δ*macAB* pBAD-*macAB* were spotted on 0.3% swimming agar supplemented with 0.02% l-arabinose and incubated for 72 h at 30°C. (A) Pictures were taken after 72 h of incubation. (B) The motility ring diameter was measured and plotted after 0, 24, and 72 h of incubation. (C) Bacterial suspension of wild-type, Δ*macAB*, and Δ*macAB*::*macAB*-FLAG mutant strains were grown in Mueller-Hinton broth in tissue culture-treated 24-well plates for 3 days at 30°C. Biofilm formation was assayed using crystal violet staining. The asterisks (in panels B and C) indicate significance in unpaired *t* tests with *P* values of <0.05. (D) Representative transmission electron microscopy (TEM) images of the wild type, Δ*macAB* mutant strain, and Δ*macAB* strain complemented with *macAB* genes in *cis*.

Inactivation of the MacAB efflux pump in Stenotrophomonas maltophilia (25) and in *S.* Typhimurium (37) resulted in decreased ability of bacteria to form biofilms. To test the role of the MacAB efflux pump in S. marcescens motility and biofilm formation, we first followed the development of a swimming colony on 0.3% swimming agar by the wild type, Δ*macAB* mutant strain, and Δ*macAB* strain bearing a wild-type copy of the *macAB* gene on the plasmid. We found that wild-type S. marcescens spreads across all available agar surfaces within 72 h of incubation (Fig. 4A). Deletion of the MacAB efflux pump significantly reduced swimming motility. The motility defect was completely reverted by complementation in *trans* (Fig. 4A and B).

Next, we allowed wild-type and Δ*macAB* mutant strains to form biofilms on a polystyrene surface in 24-well plates and evaluated biofilm formation in a crystal violet biofilm assay (38). We found that the biofilm-producing ability of the Δ*macAB* mutant strain was not altered after the first 24 h of incubation compared to wild type (Fig. 4C). However, after 48 and 72 h of incubation, biofilm formation by the mutant strain was progressively reduced compared to that of the wild type. Complementation of *macAB* deletion in *cis* led to the reversal of this mutant phenotype (Fig. 4C).

Finally, transmission electron microscopy (TEM) of wild-type S. marcescens cells showed the presence of peritrichous flagella (Fig. 4D). However, these appendages were no longer present in Δ*macAB* mutant cells. Instead, Δ*macAB* cells carried numerous short structures on the bacterial surface which may represent fimbria or defective flagella. Complementation of the Δ*macAB* mutant with a wild-type copy of *macAB* genes in *cis* restored wild-type flagellation of S. marcescens (Fig. 4D). Taken together, these data clearly indicate that the MacAB efflux pump is involved in S. marcescens motility and biofilm formation.

### The MacAB promoter is positively regulated by hydrogen peroxide.

**FIG 5:**
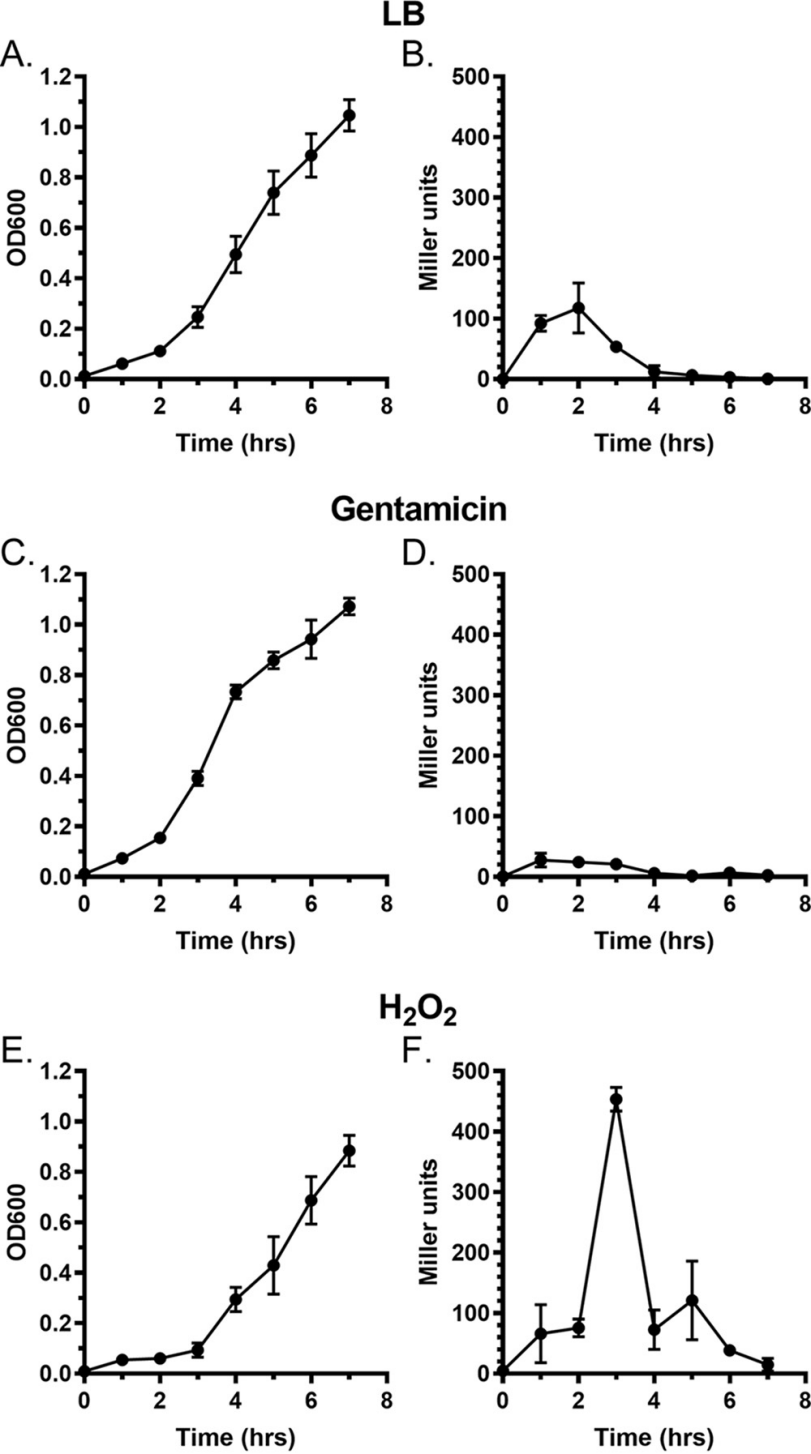
Hydrogen peroxide exposure drives the activity of the *macAB* operon promoter. (A to F) Overnight culture of S. marcescens *P_macAB_-lacZY* was subcultured at a 1/100 ratio in LB broth (A and B), LB broth containing 0.125 μg/ml gentamicin (C and D), or LB broth supplemented with 1 mM H_2_O_2_ (E and F) and incubated at 37°C with shaking. Aliquots were collected hourly and used for measurements of optical density at 600 nm (A, C, and E) or β-galactosidase activity (B, D, and F). Experiments were done on at least three separate occasions.

Conditions that modulate expression of the MacAB efflux pump in other bacterial species are currently not well understood. It is known that in the human pathogen *Salmonella* Typhimurium, the *macAB* operon is not active under standard laboratory conditions (30). On the other hand, its activity is negatively regulated by the *Salmonella* Typhimurium PhoP-PhoQ two-component system (10) and positively regulated by exposure to H_2_O_2_ (28). The opportunistic pathogen Stenotrophomonas maltophilia, when grown in LB-broth, expresses the *macABCsm* operon during log phase (25). To determine whether the S. marcescens *macAB* operon is expressed under standard laboratory conditions, we generated a mutant strain bearing a chromosomally encoded *P_macAB_-lacZY* transcriptional fusion. We found that when this strain was grown in LB broth (Fig. 5A), the promoter of the *macAB* operon was active during the first 3 h of growth in fresh medium and reached its maximum activity at 2 h (Fig. 5B), correlating with the lag phase (Fig. 5A). The activity of the MacAB promoter started to decline as S. marcescens entered log phase and remained low throughout the rest of the bacterial growth cycle.

Given that the S. marcescens Δ*macAB* mutant was more sensitive to aminoglycoside antibiotics, we sought to determine whether the presence of such drugs could influence *macAB* expression. We grew the *P_macAB_-lacZY* transcriptional reporter strain in the LB broth containing a subinhibitory concentration of gentamicin (Fig. 5C) and measured β-galactosidase activity over time (Fig. 5D). We did not detect any increase in *macAB* expression in the presence of antibiotic. In fact, β-galactosidase activity was reduced by 76% compared to growth in medium without gentamicin (Fig. 5B and D). This result might indicate that gentamicin is not a natural substrate of the MacAB efflux pump.

Finally, because the *macAB* locus in *S. Typhimurium* is induced during oxidative stress (28), we next exposed the *macAB-lacZY* reporter strain to 1 mM hydrogen peroxide (Fig. 5E). We found that expression of the MacAB efflux pump is further increased approximately 4.5-fold when H_2_O_2_ is present in the growth medium (Fig. 5F).

Collectively, these data show that the S. marcescens MacAB efflux pump is expressed during lag phase in LB broth and that its expression is regulated negatively by gentamicin and positively by the presence of hydrogen peroxide.

### MacAB is required for S. marcescens survival during oxidative stress.

**FIG 6:**
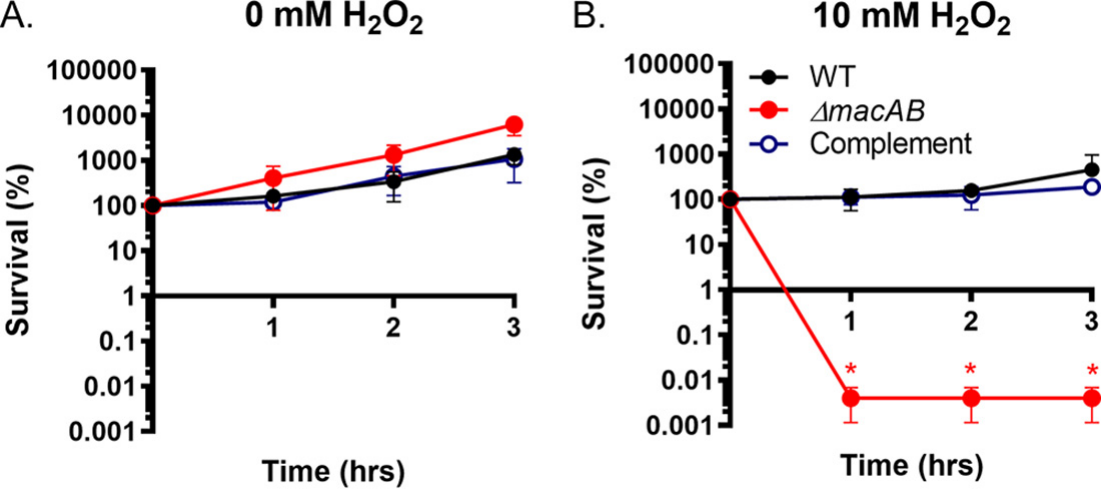
The efflux pump MacAB is required for S. marcescens survival in the presence of hydrogen peroxide. Overnight cultures of the wild type (black circles), Δ*macAB* mutant strain (red circles), and Δ*macAB* mutant strain complemented with *macAB* genes in *cis* (open blue circles) were subcultured at a 1/100 ratio in LB broth containing no peroxide (A) or 10 mM H_2_O_2_ (B). Aliquots were collected hourly, serially diluted, and plated. Data represent the survival means from at least three independent experiments and a standard error. The asterisks indicate significance in unpaired *t* tests; *P < *0.05.

In addition to their roles in drug efflux, efflux pumps play important roles in bacterial physiology. The *Salmonella* Typhimurium MacAB efflux pump is involved in virulence, intracellular survival within macrophages, and protection against an oxidative stress (27, 28). Given that peroxide exposure activates the *macAB* promoter (Fig. 5F), we evaluated the growth of wild type and Δ*macAB* mutant strains in LB broth supplemented with 10 mM H_2_O_2_ (39). In the absence of H_2_O_2_, the Δ*macAB* mutant strain grew similarly to the wild-type strain (Fig. 6A). In the presence of peroxide, wild-type S. marcescens cells stopped dividing for the first 2 h of incubation but remained viable and displayed fast growth recovery rate at later hours (Fig. 6B and data not shown). In contrast, the bacterial population of the Δ*macAB* mutant strain lost over 99.9% of its viability within the first hour of exposure to H_2_O_2_, despite the presence of active peroxide-degrading enzymes, catalases, and peroxidases (data not shown). Moreover, no restoration of the Δ*macAB* mutant strain’s growth was detected even after overnight incubation in peroxide-containing medium. Nevertheless, viability of the Δ*macAB* mutant strain in the presence of H_2_O_2_ was fully restored by providing the intact copy of *macAB* genes in *cis* (Fig. 6B). We conclude that the MacAB efflux pump is required for survival of S. marcescens during an oxidative stress.

## DISCUSSION

The *macAB* locus was found to be one of the most abundant among efflux pump genes in the metagenomic analysis of samples collected from soil, ocean, and human feces (40, 41). It was also shown by a systems biology approach that MacAB plays a key role in the resistome of pathogenic E. coli O157:H7 (42). Previously, the MacAB efflux pump was also identified in a number of Gram-negative bacteria (10, 22, 24, 43, 44). Additionally, the MacAB efflux pump is present in the core genomes of S. marcescens isolates from different ecological niches (soil, water, animals, plants, and health care-associated facilities) (45). We found that the genomic context of the *macAB* locus in the S. marcescens SM6 genome is similar to corresponding loci previously described for E. coli and *S.* Typhimurium.

The MacAB efflux pump was previously linked to resistance of E. coli and Neisseria gonorrhoeae to macrolide antibiotics (22, 24). In Acinetobacter baumannii and Klebsiella pneumoniae, however, the MacAB efflux pump is involved in resistance to the tetracycline antibiotics tigecycline and eravacycline, respectively (26, 46). Moreover, in Stenotrophomonas maltophilia, the MacABCsm efflux pump provides protection against a spectrum of antibiotics, including macrolides, aminoglycosides, and polymyxins (25). To our surprise, the deletion of *macAB* genes does not affect the sensitivity of S. marcescens to erythromycin. To further survey the role of MacAB in resistance to antibiotics, we used a commercially available panel Sensititre GN2F, which contains a number of antimicrobials in premeasured concentrations. In general, deletion of a single efflux pump with the exception of the RND (resistance-nodulation-division) efflux pump AcrAB does not affect drug sensitivity of bacteria (30, 47). Several possible explanations for this observation include redundancies in efflux pump genes and insufficient expression of the tested efflux pumps in wild-type cells under the evaluated conditions. Nevertheless, we found that deletion of *macAB* in S. marcescens altered bacterial sensitivity to the aminoglycoside antibiotic amikacin. This observation was further confirmed by the discovery that the lack of MacAB efflux pump makes bacteria more sensitive to other antibiotics of the same class, gentamicin and apramycin. Resistance to aminoglycosides required the presence of both components of the pump, suggesting the importance of the MacAB complex for protection from these drugs. This finding implies possible clinical importance of our discovery, since aminoglycosides are still the drugs of choice for treatment of infections caused by *Serratia* (32).

Polymyxins (colistin and polymyxin B) are polycationic peptides that are currently used as last-resort antibiotics for treatment of multidrug-resistant Gram-negative bacterial infections. Bacteria from the order *Enterobacterales* can develop resistance to polymyxins via acquisition of mutations in the two-component systems, which result in upregulation of the *arnBCADTEF* operon (34). In turn, this activation leads to LPS modifications that protect bacteria from polymyxins. Serratia marcescens, however, is intrinsically resistant to this class of drugs due to constitutive expression of the PhoP-dependent *arnBCADTEF* operon (35). In addition to LPS modification, resistance to polymyxins can be further modulated by efflux pumps. Contribution of RND, MFS, and MATE efflux pumps to colistin/polymyxin B resistance was reported for K. pneumoniae, S. maltophilia, A. baumannii, and *Burkholderia* species (48–54). The MacABCsm efflux pump S. maltophilia was previously linked with resistance to polymyxin B (25). Even though S. marcescens MacA and MacB proteins share only 40% and 58% amino acid identity, respectively, with the corresponding proteins in S. maltophilia, our study showed that the MacAB efflux pump contributes to polymyxin resistance of this bacterium.

Bacterial motility is one of the major factors that is essential for colonization of different surfaces. The last 2 decades of research have demonstrated the connection between the presence of various efflux pumps and motility (55–58). Inactivation of *mexGHI-opmD* genes encoding the RND-type efflux pump in P. aeruginosa PAO1 led to changes in a number of important biological functions, including reduction of swarming motility (55). Inactivation of the AcrD efflux pump in S. enterica serovar Typhimurium resulted in impaired swarming motility via changes in the expression of genes involved in fumarate metabolism (56). Similarly, deletion of genes coding for RND-type pump SmeYZ S. maltophilia resulted in a complete loss of swimming motility. Electron microscopy also showed the loss of flagella on the surface of Δ*smeYZ* cells (57). Inactivation of five of the six known efflux pumps in Acinetobacter baumannii ATCC 17978 led to reduced motility. Interestingly, two of those pumps belong to the ABC type (58).

Most opportunistic bacterial infections are associated with growth in biofilms, where bacteria are more resistant to antibiotics compared to planktonic cells (59). Efflux pumps have an important role in biofilm formation in a number of bacterial species. Efflux pump genes are upregulated in E. coli biofilms, and their inhibition in E. coli and Klebsiella pneumonia severely impact biofilm formation (60, 61). Moreover, six different efflux pumps in E. coli K-12 contribute to biofilm formation (62). Similarly, deletion of *S.* Typhimurium efflux pump genes or inactivation of active efflux through chemical inhibition implicated all tested pumps in biofilm formation (37). In agreement with these data, our results show that the MacAB efflux pump of S. marcescens SM6 is needed for optimal swimming motility and biofilm formation.

With the exception of a few housekeeping efflux pumps, i.e., AcrAB in E. coli and *S.* Typhimurium, most efflux pumps are expressed at a low basal level in the absence of inducing signals (30). Similarly, expression of the MacAB efflux pump in *S*. Typhimurium is low in the standard laboratory medium (28). Our results indicate that in S. marcescens, the *macAB* gene promoter is active only during the initial hours of growth in LB broth.

Efflux pump genes are often controlled by local repressors and global regulators, including the two-component systems. Local repressors usually belong to the TetR, MarR, or MerR family of transcriptional factors. For a number of efflux pumps, these repressor genes are located in proximity to efflux pump genes (63). However, no such genes are present in the vicinity of *macAB* genes in the S. marcescens genome (29). Additionally, expression of efflux pumps can be controlled by global regulators. For instance, expression of the E. coli AcrAB efflux pump is modulated by XylS/AraC family regulators MarA, Rob, and SoxS in response to different environmental stimuli, while in *S.* Typhimurium, this pump is also regulated by RamA, an AraC family transcriptional regulator (64). The MacAB efflux pump in *S.* Typhimurium is negatively regulated by global regulator PhoP. The mechanism by which the MacAB efflux pump is regulated in S. marcescens is currently unknown. The analysis of the nucleotide sequence upstream of the *macAB* operon using Virtual Footprint promoter analysis v.3 (65) indicated the presence of putative binding sites for global regulators ArcA and OxyR (data not shown). Both transcription factors are known to positively regulate the expression of genes encoding components of the oxidative pathway (66, 67). While the exact mechanism of MacAB regulation in S. marcescens is yet to be defined, our results clearly indicate that activation of the *macAB* promoter is peroxide-dependent.

In addition to its well-characterized role in protection of bacteria against antimicrobials, drug efflux pumps play important roles in other cellular processes. For example, the AcrAB efflux pump is involved in the secretion of bile in E. coli and S. enterica serovar Typhimurium. Induction of the efflux pump by bile is one of the key factors mediating bacterial resistance to lipophilic antibiotics (68, 69). Moreover, the expression of *acrAB* is upregulated in E. coli in the presence of sodium chloride and ethanol (70). The MacAB efflux pump was linked to secretion of heat-stable enterotoxin II produced by enterotoxigenic E. coli and to the export of the E. coli heme precursor protoporphyrin PPIX (23, 71). More recently, MacAB was shown to be involved in survival of *S.* Typhimurium during oxidative stress through secretion of linearized siderophore enterobactin (27, 28). Interestingly, production of S. marcescens siderophores is affected by deletion of the MacAB efflux pump (72). Our results further demonstrate that the role of the MacAB efflux pump in protection from oxidative stress is conserved across different bacterial species. Furthermore, the function of this pump in S. marcescens is essential for bacterial survival, despite the presence of peroxide-degrading enzymes, catalases, and peroxidases. Individuals with chronic granulomatous disease (CGD) are particularly prone to *Serratia* infections due to impaired production of reactive oxygen species by neutrophils and macrophages (73). Identification of the MacAB efflux pump as a plausible target for inhibition could, therefore, improve therapy for these patients.

In conclusion, our study implicates the S. marcescens MacAB efflux pump in protection from aminoglycoside antibiotics and polymyxins, the modulation of bacterial motility, and biofilm formation, and it illustrates its essential role in bacterial survival during oxidative stress. Further elucidation of the specific role that the S. marcescens MacAB efflux pump plays in protection from peroxide-mediated damage will provide new and important insights into the intricate mechanisms of bacterial pathogenicity.

## MATERIALS AND METHODS

### Bacterial strains, media, and growth.

All S. marcescens strains used in this study are listed in Table 3. The Δ*macAB* mutant strain was generated by lambda red homologous recombination (74, 75) in nuclease-deficient, restrictionless S. marcescens strain TT392 (76), and the mutation was then moved into S. marcescens SM6 by bacteriophage ΦOT8 transduction (77, 78). Strains were routinely grown in LB broth (10 g/liter Bacto-Tryptone, 5 g/liter yeast extract, 5 g/liter NaCl) or Mueller-Hinton broth (BD Difco). When needed, antibiotics were used at the following concentrations: 20 mg/liter chloramphenicol, 50 mg/liter kanamycin, 100 mg/liter carbenicillin. Bacteria were grown at 37°C with shaking (200 rpm) unless stated otherwise.

**TABLE 3:** Strain list

Strain	Description	Reference or source
LMB1	S. marcescens SM6 wild type	74
LMB28	S. marcescens TT392 Δ*macAB*::Cm^R^	74
LMB71	S. marcescens SM6 Δ*macAB*::Cm^R^	This study
LMB197	S. marcescens TT392 *P_macAB_-lacZY*	This study
LMB406	S. marcescens SM6 *P_macAB_-lacZY*	This study
LMB184	S. marcescens TT392 *macB-*6xHis, Kan^R^	This study
LB485	S. marcescens TT392 *macA-*FLAG, Cm^R^	This study
LMB163	S. marcescens TT392 *macB-*FLAG, KanR	This study
LMB456	S. marcescens SM6 Δ*macAB*::*macAB*-FLAG, Kan^R^	This study
LMB430	S. marcescens SM6 pBAD30, Amp^R^	This study
LB515	S. marcescens SM6 pWSK29, Amp^R^	This study
LMB436	S. marcescens SM6 Δ*macAB*::Cm^R^ pBAD30, Amp^R^	This study
LMB448	S. marcescens SM6 Δ*macAB*::Cm^R^ pBAD30-*macAB*-6xHis, Kan^R^	This study
LB897	S. marcescens SM6 Δ*macAB*::Cm^R^ pWSK29, Amp^R^	This study
LB899	S. marcescens SM6 Δ*macAB*::Cm^R^ pWSK29-*macA*-FLAG, Cm^R^	This study

### Plasmid construction.

The complementation plasmid carrying intact *macAB* genes with C-terminal 6×His tag was generated as follows: S. marcescens strain with chromosomal macB-6×His fusion (LMB184) was generated using the previously established protocol (74, 79) with primers macB-6×His-FWD 5′-TGCCGCGCGGCTGAATCCGATCGATGCGCTGGCGCGCGAGCACCACCATCATCACCATTAGT-3′ and macB-tagging-REV 5′-TGCCAGCCGCCGTGTGACTGGCATTTTTTATGCCTTTTACTATGAATATCCTCCTTAG-3′ and the template plasmid pSUB7 (79). A DNA fragment containing the full-length open reading frame with 247 bp upstream and 183 bp downstream of *macAB* was amplified using genomic DNA from LMB184 by PCR with macA-FWD-SacI 5′-AGCGAGCTCTGACATGATGAAATCCTT-3′ as a forward primer and macB-REV-KpnI 5′-ATGGTACCAAGGCGGGCCAGCAGGTC-3′ as a reverse primer. PCR product was digested with SacI and KpnI (New England Biolabs) and ligated into pBAD30 vector (Invitrogen) previously digested with the same enzymes. Clones with the correct insert were confirmed by restriction digestion and sequencing.

The S. marcescens strain with chromosomal macA-FLAG fusion (LB485) was generated with primers macA-FLAG-FWD 5′-CGATGAGGTGATCGTCAGCCGCGGCGGCGTGGAGGCCGGCGACTACAAAGATGACGACGATAAATAG-3′ and macA-tagging-REV 5′-TAGCTGCGGCGAATGCCGTTCAGCTGCAACAGCGCCGCCATATGAATATCCTCCTTAG-3′ and the template plasmid pSU313 using the previously established protocol (74, 79). The resulting strain was used for PCR amplification of the *macA* coding sequence with 247 bp upstream and 119 bp downstream using primers macA-FWD-SacI as a forward primer and macA-REV-KpnI 5′-ATGGTACCCATGATCGCCACCATTTC-3′ as a reverse primer. PCR product was digested with SacI and KpnI (New England Biolabs) and ligated into pWSK29 vector (80) previously digested with the same enzymes. Clones with the correct insert were confirmed by restriction digestion and sequencing.

### Complementation of *macAB* locus deletion in *cis*.

The S. marcescens strain with chromosomal *macB*-FLAG fusion (LMB163) was generated using a previously established protocol (74, 79). Briefly, primers macB-FLAG-FWD 5′-TGCCGCGCGGCTGAATCCGATCGATGCGCTGGCGCGCGAGGACTACAAAGATGACGACGATAAATAG-3′ and macB-tagging-REV were used to amplify antibiotic-resistant cassette from the template plasmid pSU312 (79). Genomic DNA from LBM163 was used to amplify a DNA fragment by PCR using primers macA-FWD-SacI and macB-REV-KpnI. The resulting linear DNA fragment was used for lambda red-mediated homologous recombination to replace the chloramphenicol resistance cassette in the Δ*macAB* mutant strain (LMB28) with the full-length *macAB* coding sequence bearing FLAG tag and resistance to kanamycin. The mutant construct was moved into the S. marcescens SM6 strain background by bacteriophage ΦOT8 transduction (77, 78). The resulting clones were confirmed by PCR.

### Growth in erythromycin.

Overnight cultures of wild-type, Δ*macAB*, and Δ*macAB pmacAB* mutant strains were subcultured at a 1:100 ratio in fresh Mueller-Hinton (MH) broth and incubated at 35°C with shaking (200 rpm) until each bacterial suspension reached turbidity equal to 0.5 McFarland standards. Each resulting culture was further diluted and used to inoculate a 96-well dish containing MH broth without antibiotic or MH broth supplemented with 128 and 256 mg/liter erythromycin (Chem-Impex), respectively, to a final concentration of approximately 5 × 10^5^ CFU/ml. The 96-well dish was sealed with Breathe-Easy membrane (Diversified Biotech) to reduce evaporation and incubated for 16 h at 35°C. The optical density at 600 nm (OD_600_) was measured using a spectrophotometer (BioTek Synergy HTX). Experiments were done in triplicate.

### MIC testing using Sensititre.

The MICs for wild-type, Δ*macAB* mutant, and complementing strains (Δ*macAB*::*macAB*-FLAG, LMB456) were determined with a microdilution method using Sensititre GN2F plates (Thermo Scientific) according to the manufacturer’s recommendations. Briefly, Sensititre panels are plastic microtiter plates containing dried antimicrobial agents in premeasured quantities. Fresh colonies of the wild type, Δ*macAB*, and the complementing mutant strain were transferred from the agar plate into corresponding tubes containing 11 ml cation-adjusted MH broth. The resulting bacterial suspensions were used to inoculate Sensititre GN2F plates. Microtiter plates were incubated for 18 h at 35°C. The MIC was defined as the lowest concentration of antimicrobial agent that inhibited visible growth of bacteria. Experiments were done on at least three separate occasions.

### Antibiotic susceptibility testing using disk diffusion assay.

The susceptibilities of wild-type and Δ*macAB* mutant strains were determined by disk diffusion assay on Mueller-Hinton agar (BD Difco) according to CLSI guidelines (www.clsi.org). Briefly, a fresh colony of each tested strain was picked from the agar plate, resuspended in 0.9% saline, and adjusted to 0.5 MacFarland standards. Bacterial suspension was then streaked on the Mueller-Hinton agar using a sterile cotton-tipped wooden applicator. Disks with antibiotics (Research Center for Pharmacotherapy, Saint Petersburg, Russia) were placed on the agar surface. Plates were incubated for 18 h at 35°C. Experiments were done in duplicate on at least three separate occasions.

### Growth in gentamicin, apramycin, colistin, and polymyxin B.

Overnight cultures of wild-type, Δ*macAB*, Δ*macAB pmacA*, and Δ*macAB pmacAB* mutant strains were subcultured at a 1:100 ratio in fresh MH broth and incubated at 35°C with shaking (200 rpm) until each bacterial suspension reached turbidity equal to 0.5 McFarland standards. Each resulting culture was further diluted and used to inoculate a 96-well dish containing MH broth without antibiotic or MH broth supplemented with 0.5, 1, and 2 mg/liter gentamicin (Sigma-Aldrich), 4, 8, and 16 mg/liter apramycin (Ambeed), 512, 1,024, and 2,048 mg/liter colistin (Chem-Impex), or polymyxin B (Sigma-Aldrich), respectively, to a final concentration of approximately 5 × 10^5^ CFU/ml. The 96-well dish was sealed with a Breathe-Easy membrane (Diversified Biotech) to reduce evaporation and was incubated for 16 h at 35°C. The optical density at 600 nm (OD_600_) was measured using a spectrophotometer (BioTek Synergy HTX). Experiments were done in triplicate.

### Swimming motility assay.

Cultures of wild type carrying an empty pBAD30 plasmid and the Δ*macAB* pBAD30 and Δ*macAB* pBAD30-*macAB*-6×His mutant strains were grown overnight in LB broth supplemented with carbenicillin (RPI Corp.) at 37°C with shaking (200 rpm). Cultures were then normalized by the OD_600_, and 5 μl of each strain was spotted on 0.3% swimming agar (81) supplemented with 0.02% l-arabinose to induce expression of the *macAB* operon. Plates were incubated for 72 h at 30°C. The experiment was done in triplicate.

### Biofilm formation.

The wild type and the Δ*macAB* and Δ*macAB*::*macAB*-FLAG mutant strains were grown in 3 ml MH broth in sterile tissue culture-treated 24-well plates (Eppendorf, Germany) for 3 days at 30°C without shaking. Planktonic bacteria were removed by aspiration; wells were then washed with 3 ml of sterile 0.9% saline. Biofilms were air-dried and stained with 600 μl of 0.1% crystal violet (Dia-M, Russia) for 15 min at room temperature. Subsequently, the dye was removed, and the wells were washed 3 times with sterile 0.9% saline. The wells were allowed to dry, and the stained biofilms were solubilized with 600 μl of ethanol. To evaluate biofilm formation, 300 μl from each well was used to measure the optical density (OD) at 595 nm using a microtiter-plate reader (Bio-Rad iMark Microplate Absorbance Reader, Japan). The experiments were performed in triplicate.

### Transmission electron microscopy (TEM).

The wild type, the Δ*macAB* mutant, and the complementing Δ*macAB*::*macAB*-FLAG (LMB456) mutant strains were grown overnight in LB broth with appropriate antibiotics at 30°C with shaking. Cells were pelleted by centrifugation at 4,000 rpm for 5 min (Hermle Z326K, Germany) and washed twice in phosphate buffer (0.06 M Na_2_HPO_4_ × 7H_2_O, 0.04 M NaH_2_PO_4_ × H_2_O, pH 7.0) by gentle pipetting. Then, 5 ml of bacterial suspensions were applied on the Formvar/carbon-coated copper grid (3 mm) and allowed to dry at room temperature. The grid was placed in a transmission electron microscope (Hitachi HT7700 Exalens). Analysis was done at an accelerating voltage of 100 kV.

### β-Galactosidase activity.

The chromosomal *P_macAB_-lacZY* fusion construct was generated using an established protocol (82). Briefly, the TT392 Δ*macAB*::Cm^R^ mutant strain (LMB28) was transformed with pCP20 (75) plasmid to remove the antibiotic resistance cassette. The Δ*macAB*::FRT pCP20 mutant strain was then electroporated with *lacZY* transcriptional fusion plasmid pKG137 (82). The *lacZY* fusion construct was then integrated into the Flp target sequence downstream of the *macAB* promoter by Flp-mediated recombination. Positive clones were selected on LB/Kan/X-Gal (5-bromo-4-chloro-3-indolyl-β-d-galactopyranoside) plates at 37°C. The final construct was transferred into S. marcescens SM6 genetic background by ΦOT8 bacteriophage transduction (LMB71).

Overnight cultures of wild-type or chromosomal *macAB-lacZY* fusion strain (LMB71) were subcultured 1/100 in fresh LB broth or in LB broth containing either 0.125 mg/liter gentamicin (Gibco) or 1 mM H_2_O_2_ (AppliChem). Cultures were grown at 37°C with shaking. Aliquots for OD_600_ measurements of both the wild type and the reporter strain cultures, as well as aliquots for β-galactosidase assay were taken hourly. β-Galactosidase was assayed with a modified Miller protocol (83). In brief, 20 μl of the bacterial culture at each given time point was mixed with 80 μl of permeabilization solution (100 mM Na_2_HPO_4_, 20 mM KCl, 2 mM MgSO_4_, 0.8 mg/ml CTAB [hexadecyltrimethylammonium bromide], 0.4 mg/ml sodium deoxycholate, 5.4 μl/ml β-mercaptoethanol) and incubated for 30 min at 30°C. Then, 600 μl of substrate solution (60 mM Na_2_HPO_4_, 40 mM NaH_2_PO_4_, 1 mg/ml *o*-nitrophenyl-β-d-galactoside [ONGP, Thermo Scientific], 2.7 μl/ml β-mercaptoethanol) was added to each tube and incubated at 30°C until color development, followed by the addition of 700 μl of stop solution (1 M Na_2_CO_3_). Samples were centrifuged for 5 min at maximum speed (Hermle Z326K), followed by OD_420_ measurements. β-Galactosidase activity was expressed in Miller units. Miller units were calculated as ([OD_420_/OD_600_ · *t* · *v*] · 1,000), where *t* is the reaction time in minutes and *v* is the volume of culture assayed in milliliters (*v* = 0.02). β-Galactosidase activity of wild-type culture was determined in parallel and was subtracted from the values of the reported strain to account for the background levels of enzymatic activity. Results were expressed as a function of time. The experiment was done on at least three separate occasions.

### Sensitivity of S. marcescens strains to hydrogen peroxide.

Overnight cultures of wild type, Δ*macAB* mutant, and the complementing Δ*macAB*::*macAB*-FLAG (LMB456) mutant strains were subcultured at 1 to 100 ratios in fresh LB broth with or without 10 mM H_2_O_2_ (AppliChem). The resulting cultures were incubated at 37°C with shaking. Aliquots were collected hourly, serially diluted, and plated for CFU determination. Results were expressed as percent survival [CFU(*t*_n_)/CFU(*t*_0_)] · 100 over time. Experiments were done on at least three separate occasions.

### Data analysis.

Statistical significance was determined using the unpaired *t* test with Welch correction; *P < *0.05. Analyses were performed using GraphPad Prism v.9.0.0.
